# Explicit Features Versus Implicit Spatial Relations in Geomorphometry: A Comparative Analysis for DEM Error Correction in Complex Geomorphological Regions

**DOI:** 10.3390/s26061995

**Published:** 2026-03-23

**Authors:** Shuyu Zhou, Mingli Xie, Nengpan Ju, Changyun Feng, Qinghua Lin, Zihao Shu

**Affiliations:** State Key Laboratory of Geohazard Prevention and Geoenvironment Protection, Chengdu University of Technology, Chengdu 610059, China; zhoushuyu@stu.cdut.edu.cn (S.Z.); jnp@cdut.edu.cn (N.J.); 2023050233@stu.cdut.edu.cn (C.F.); linqinghua@stu.cdut.edu.cn (Q.L.); 2023020295@stu.cdut.edu.cn (Z.S.); Badong National Observation and Research Station of Geohazards, China University of Geosciences, Wuhan 430074, China; State Key Laboratory of Hydraulics and Mountain River Engineering, Sichuan University, Chengdu 610059, China

**Keywords:** DEM error correction, ICESat-2, geospatial AI, model comparison, Explainable AI (XAI), Graph Neural Networks (GNNs)

## Abstract

**Highlights:**

**What are the main findings?**

- Severe Scale Mismatch: GNN-XGBoost hybrid models yield negligible accuracy gains (<0.05 m) at a nearly 18-fold computational cost. The vast spatial baseline of ICESat-2 footprints (averaging ~485 m) severely violates the dense connectivity assumption of GNNs, inducing predictable “over-smoothing”.
- Mechanism Decoupling & ID Chaos: A critical divergence in decision logic is uncovered: XGBoost strictly isolates deterministic physical drivers (e.g., Aspect) with near-perfect stability (ρ > 0.97). Conversely, GNNs exhibit severe “Attribution Stochasticity” (ρ≈ 0.63–0.77), acting as opportunistic “residual-dependent latent feature learners” that fit local noise.

**What are the implications of the main findings?**

- Physics Trumps Geometry: For geospatial regression tasks relying on sparse supervision, explicit physical feature engineering offers superior interpretability and transferability compared to “Black Box” geometric deep learning, challenging the “complexity is better” assumption.
- New Evaluation Standard: The identification of “stable prediction, drifting explanation” (Underspecification) underscores a critical imperative: Geo-AI research must prioritize “Explanatory Stability” as a prerequisite for scientific inference, rather than solely pursuing marginal accuracy gains.

**Abstract:**

Global Digital Elevation Models (DEMs) exhibit systematic biases constrained by acquisition geometry and surface penetration. This study aims to evaluate whether the increasing complexity of geometric deep learning (e.g., Graph Neural Networks, GNNs) is justified by performance gains over established feature engineering paradigms (e.g., XGBoost) under the constraints of sparse altimetry supervision. We established a rigorous comparative framework across four mainstream products—ALOS World 3D, Copernicus DEM, SRTM GL1, and TanDEM-X—using Sichuan Province, China, as a representative natural laboratory. Our results reveal a fundamental scale mismatch (where the ~485 m average spacing of sampled altimetry footprints dwarfs the local terrain resolution): despite their topological complexity, Hybrid GNN models fail to establish a statistically significant accuracy advantage over the systematically optimized XGBoost baseline, demonstrating RMSE parity. Mechanistically, we uncover a critical divergence in decision logic: XGBoost relies on a stable “Physics Skeleton” consistently dominated by deterministic features (terrain aspect and vegetation density), whereas GNNs exhibit severe “Attribution Stochasticity” (ρ ≈ 0.63–0.77). The GNN component acts as a residual-dependent latent feature learner rather than discovering universal topological laws. We conclude that for geospatial regression tasks relying on sparse supervision, “Physics Trumps Geometry.” A “Feature-First” paradigm that prioritizes robust, domain-knowledge-based physical descriptors outweighs the indeterminate complexity of “Black Box” architectures. This study underscores the imperative of prioritizing explanatory stability over marginal accuracy gains to foster trusted Geo-AI.

## 1. Introduction

Digital Elevation Models (DEMs), as the digital representation of the Earth’s surface morphology, serve as an indispensable foundational dataset supporting a wide range of Earth science applications, including hydrological modeling [1], geohazard assessment [2], ecosystem research [3], and global change monitoring [4]. In recent years, the proliferation of various global open-source DEM products, such as SRTM GL1 DEM [5], TanDEM-X EDEM [6,7], ALOS World 3D [8], and Copernicus DEM [9], has significantly advanced research in related fields. However, it is crucial to clarify that most of these products are essentially Digital Surface Models (DSMs), representing the reflective surface of canopy and structures rather than the bare terrain [10,11]. The data acquisition and processing steps for these products introduce inherent systematic and random errors. These biases are constrained by multiple factors, including sensor type (e.g., InSAR, optical photogrammetry), terrain complexity, surface cover (especially vegetation), and atmospheric conditions [12]. Recent global validation studies highlight that these vertical errors are not randomly distributed but exhibit strong correlations with geomorphometric variables like slope and aspect [13]. Consequently, the high-precision error correction of these datasets remains a critical research priority.

The advent of high-precision global altimetry missions, particularly the successful operation of NASA’s Ice, Cloud, and Land Elevation Satellite-2 (ICESat-2), has provided unprecedented opportunities for DEM error correction. ICESat-2’s advanced photon-counting lidar system delivers globally distributed ground elevation control points with centimeter-level vertical accuracy [14,15]. While the use of high-resolution airborne LiDAR DTMs is often considered the gold standard for validation, such data are often scarce or unavailable in large-scale, complex terrains like Western China. In this context, ICESat-2 serves as the most viable “ground truth” alternative. Utilizing these high-fidelity laser points as “ground truth,” per-point error assessment and correction of DEMs has become the mainstream technical approach in this domain [16,17,18].

The methodology for DEM error correction has witnessed a paradigm shift. Early efforts primarily relied on global trend surface analysis and classical geostatistical interpolation (e.g., Kriging), which explicitly models the spatial autocorrelation of errors. Despite the rapid development of new algorithms, Kriging remains a robust and widely used benchmark in recent high-precision DEM assessments [19]. However, the field has progressively transitioned toward the contemporary era of data-driven machine learning, driven by the need to capture more complex non-linear patterns. Currently, a widely validated and highly successful paradigm is Machine Learning driven by Feature Engineering (FE-ML). Researchers consistently find that DEM errors correlate strongly with multiple quantifiable geomorphometric and geophysical factors. In this study, we define this paradigm as modeling based on “Explicit Physical Features”—variables governed by clear physical laws, such as slope (gravitational potential), aspect (solar illumination angle), and vegetation cover fraction. By constructing such an explicit feature set and applying Gradient Boosting Decision Tree (GBDT) models, such as XGBoost [20] or LightGBM [21], to learn the complex non-linear relationship between these features and the elevation error, exceptional correction performance is achieved [22,23,24]. The essence of this paradigm lies in encoding extensive geo-scientific domain knowledge into structured, numerical features that the model can directly utilize.

Nevertheless, the FE-ML paradigm traditionally treats error points as independent samples. From a geostatistical perspective, this neglects the inherent “spatial non-stationarity” and anisotropy of terrain errors, where error distribution varies directionally and clusters spatially [25]. To overcome this inherent constraint, an emerging paradigm from the field of deep learning—Geometric Deep Learning, specifically Graph Neural Networks (GNNs)—has demonstrated immense potential [26,27,28]. Unlike FE-ML, GNNs rely on “Implicit Spatial Relations”—the latent topological dependencies between adjacent pixels learned through message-passing mechanisms [29]. From a geomorphometric perspective, elevation errors are rarely isolated point-wise anomalies; instead, they exhibit strong spatial autocorrelation [30]. For instance, systematic elevation biases—such as those caused by radar shadowing or canopy penetration limits—often act as artificial dams, blocking overland flow paths and causing severe distortions in downstream hydrological drainage networks [31]. These topographically constrained biases create a continuous chain of spatially dependent errors across the landscape. Theoretically, by structuring ICESat-2 points as a geospatial graph, GNNs should capture spatially dependent error patterns missed by FE-ML models [32,33,34].

Despite the theoretical advantages of GNNs, a notable gap remains in the literature. While contemporary machine learning models have significantly improved global error metrics like RMSE, their stability and interpretability in a geospatial context remain a notorious “black box.” It is often unclear whether these deep learning architectures are genuinely learning universal topographic laws or merely overfitting to spatial noise. Therefore, a pivotal question arises: Is the increasing complexity of these deep learning models justified by their marginal performance gains? In a scenario where feature engineering is already highly refined, can the “Implicit Spatial Relations” of GNNs genuinely deliver a performance advantage over state-of-the-art models that depend on “Explicit Physical Features”? Or, does a robust physical feature set effectively “short-circuit” the need for complex spatial convolution?

To address these critical scientific questions, this study designs and executes a comprehensive comparative experimental framework. Instead of a dispersed global analysis, we focus on the structurally complex terrain of Sichuan Province, China. This region serves as a unique “natural laboratory” containing diverse geomorphological units (including basins, high mountains, and plateaus) within a consistent tectonic framework. We conduct a rigorous performance and mechanistic comparison of multiple hyperparameter-tuned GNN variants—including the foundational GraphSAGE [35], the advanced Graph Attention Network (GAT) [36], and a GNN-XGBoost hybrid model—against an equally and systematically optimized XGBoost [20] strong baseline across four mainstream DEM products. To ensure rigorous validation across different spatial supports, we employ a strict point-to-surface matching strategy to align ICESat-2 footprints with DEM grids.

To systematically open this “black box,” the specific objectives of this study are threefold: (1) to evaluate whether the topological complexity of GNNs yields statistically significant accuracy improvements over optimized FE-ML models under sparse supervision; (2) to decode the internal decision-making mechanisms of both paradigms to verify if their logic aligns with physical geomorphological laws; and (3) to assess the explanatory stability of these models to prevent underspecification risks in Geo-AI.

The primary contributions of this research are as follows:

(1) We present the first systematic comparison of these two prevailing paradigms for DEM error correction, revealing a stable and, to some extent, counter-intuitive performance hierarchy where “Explicit Physics” rivals “Implicit Geometry.”
(2) Through the use of multi-source explainability tools (SHAP [37] and GNNExplainer [38]), we uncover for the first time the fundamental differences in the decision-making logic between these two modeling paradigms.
(3) We reveal a decoupling between predictive stability and explanatory stability in GNN-based models, highlighting the risk of “Categorical Overfitting”.
(4) Drawing upon these findings, we offer a critical and broadly applicable reflection on Pareto Optimality in model selection strategies within the geospatial AI domain.

## 2. Study Area and Data

### 2.1. Study Area

**Figure 1:**
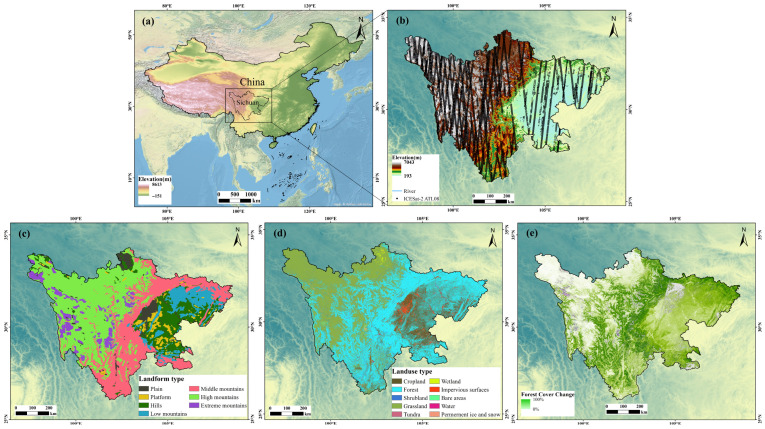
The study area and ICESat-2 ATL08 orbital tracks. (**a**) Location of the study area. (**b**) Overall, ATL08 tracks, where black dots (which may appear as solid lines due to high data density) represent ICESat-2 laser footprints on the ground. (**c**) Detailed landform classification of the study area. (**d**) Land cover types. (**e**) Spatial distribution of forest canopy cover density (%) in 2015.

Sichuan Province, located in southwestern China, serves as the transitional zone between the Tibetan Plateau and the middle-lower Yangtze River Plain (Figure 1a). The region spans approximately 97°21′ to 108°33′ E longitude and 26°03′ to 34°19′ N latitude, covering an area of about 486,000 km^2^. The topography of Sichuan is exceptionally diverse, encompassing a wide range of landforms including high mountains, deep valleys, rolling hills, and expansive basins (Figure 1c). In addition to the complex topography, the region exhibits diverse land cover types, primarily dominated by extensive forests and grasslands (Figure 1d). The dense forest canopy and its coverage variations (Figure 1e) significantly influence radar signal penetration, introducing typical scattering biases in conventional DEMs. This complex and varied terrain, coupled with diverse surface covers, makes the province an ideal natural laboratory for assessing the performance of different DEM products under diverse conditions. Consequently, the region is highly effective for a robust evaluation of DEM product accuracy, supported by the extensive spatial coverage of ICESat-2 ATL08 orbital tracks across the entire area (Figure 1b).

### 2.2. Datasets

This study evaluates four widely used global Digital Elevation Models (technically Digital Surface Models [10]): ALOS World 3D (v3.2) [39,40], Copernicus DEM [41], SRTM GL1 (V003) [42,43], and TanDEM-X EDEM [6,7]. The fundamental characteristics, acquisition periods, and baseline accuracies of these products are summarized in Table 1. To provide high-precision reference elevations, we utilized the ATL08 Land and Vegetation Height product from the ICESat-2 mission [44]. The ATL08 data, which offers global coverage and a validated vertical accuracy of approximately 0.75 m [15], serves as the ground truth benchmark for our correction models.

Furthermore, to construct a comprehensive set of predictive attributes for the machine learning models, three auxiliary datasets were integrated. These include the 10 m GLC_FCS10 for land cover classification [45], the 30 m GFCC30TC for vegetation canopy cover [46,47,48], and a 1:1,000,000 scale geomorphological map for landform classification [49].

Detailed classification schemes for land cover and landform types are provided in Appendix A (Table A1) and Appendix B (Table A2), respectively.

### 2.3. Data Preprocessing

The vertical accuracy of DEMs is predominantly assessed by comparison against higher-precision reference data [50]. While global open-source DEMs are technically Digital Surface Models (DSMs) [10,11], we follow established academic convention and refer to them as DEMs. We selected the high-fidelity ICESat-2 ATL08 product as the benchmark to systematically evaluate four global DEMs: ALOS World 3D, Copernicus DEM, SRTM GL1, and TanDEM-X EDEM. For the sake of brevity, the four global DEMs will be referred to by the acronyms AW3D, CopDEM, SRTM, and TanDEM, respectively.

Using Python (v3.11.9), a total of 2,253,544 initial laser footprints (acquired in 2024) were extracted from the HDF5 format of the raw ATL08 data products within the study area. ATLAS photon data are susceptible to ranging errors induced by atmospheric attenuation, surface reflectivity, and terrain slope (particularly during daytime observations) [51,52].

**Figure A1:**
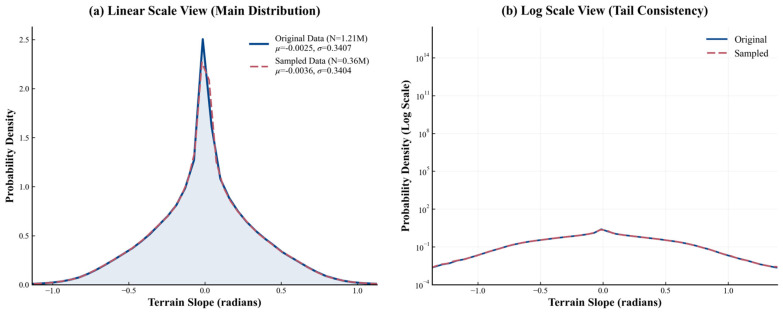
Sampled dataset.

We therefore applied filtering thresholds based on established standards [53], retaining only points that met the criteria: cloud_flag_atm < 2, and night_flag = 1. After this filtering process, 1,213,113 valid control points that met the experimental criteria were obtained. To balance computational efficiency with data representativeness for GNN graph construction, a stratified randomized sampling strategy retained 30% of the data (363,934 points). Kernel Density Estimation (KDE) analysis (Appendix C, Figure A1) confirmed this sampling strictly preserved the original topographic distribution.

To address the inherent disparities in temporal acquisition and vertical datums among the diverse datasets, a standardized harmonization protocol was applied. A common challenge in global DEM evaluation is the temporal mismatch between legacy acquisitions (e.g., SRTM in 2000) and recent altimetry data. We proceeded under the geomorphometric assumption that natural bare-earth topography remains macroscopically stable over decadal scales. Any temporal elevation discrepancies—primarily driven by dynamic land-cover changes (e.g., forest growth or harvesting)—are dynamically accounted for in our modeling framework by explicitly incorporating the vegetation canopy cover parameter (GFCC30TC) as a predictive feature. Regarding vertical datums, ICESat-2 employs unprojected WGS84 ellipsoidal heights [54]. To ensure physical consistency, CopDEM orthometric heights were explicitly converted to WGS84 ellipsoidal heights using the standard EGM2008 gravitational model equation:

(1) $$H_{elip}=H_{ortho}+G,$$

where $H_{elip}$ is the ellipsoidal height, $H_{ortho}$ is the orthometric height, and the geoid undulation (G) was obtained from the widely validated “WGS84.img” grid provided by ArcGIS 10.8 [55].

Following datum unification, rigorous spatial alignment was executed to ensure precise correspondence between the discrete footprints and continuous grids. This alignment was performed via a geographic coordinate co-registration method [56] in the native WGS84 environment to minimize reprojection errors. We adopted a “Center-Point Bilinear Interpolation” method to extract DEM features exactly at the center coordinates of each ICESat-2 footprint. For derivative calculations involving horizontal distances, geodesic formulas were strictly employed.

**Figure 2:**
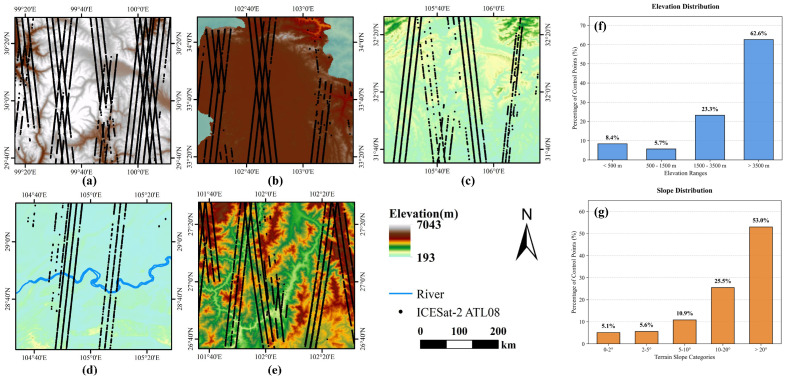
Local spatial alignment and geomorphological representativeness of the filtered ICESat-2 footprints. Panels (**a**–**e**) illustrate the detailed track coverage and point distribution across five typical geomorphological units within the study area: (**a**) high mountains, (**b**) plateaus, (**c**) hills, (**d**) plains, and (**e**) valleys. Panels (**f**,**g**) quantitatively verify the topographical representativeness of the final dataset, displaying the percentage of control points across different elevation ranges and terrain slope categories, respectively.

After masking no-data values and special features (water/ice), the final aligned dataset comprised 346,592 valid control points. To explicitly demonstrate the geomorphological representativeness of this sparse supervision dataset, we summarize its topographic distribution alongside local spatial alignments in the redesigned Figure 2. The dataset captures the profound topographic gradient of Sichuan Province effectively: as indicated in the statistical histograms (Figure 2f), while 8.4% of the points fall within low-altitude basins (<500 m), a dominant 62.6% are distributed across high-altitude plateaus and mountainous regions (>3500 m). Crucially, the slope distribution (Figure 2g) confirms that the sampling is not biased towards flatlands; 53.0% of the control points are situated on challenging, rugged terrains with slopes greater than 20°. This robust representation directly ensures the objective evaluation of model performance under extreme and diverse geomorphological conditions.

## 3. Methods

**Figure 3:**
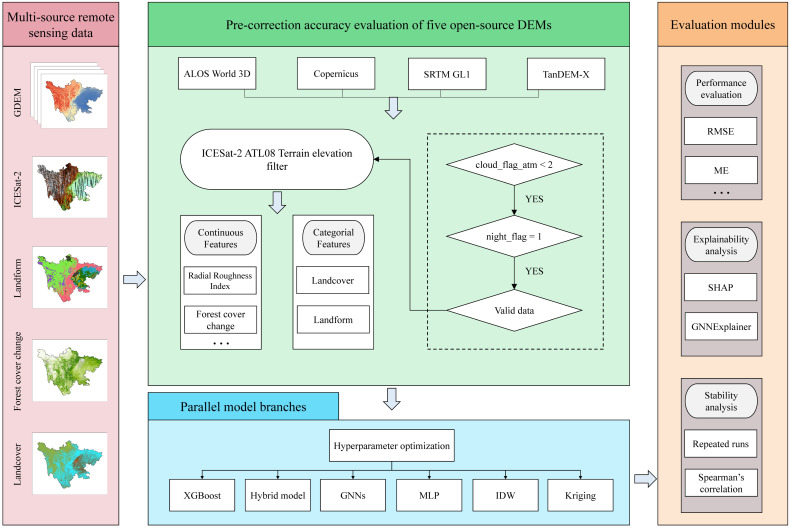
The general workflow of this research.

The detailed technical workflow of this study, systematically progressing from data preprocessing to parallel model construction and comprehensive evaluation, is explicitly outlined in Figure 3.

### 3.1. Feature Engineering

To comprehensively capture the drivers of DEM error and support both conventional machine learning and geometric deep learning paradigms, we engineered a specific set of 10 auxiliary features alongside the raw ICESat-2 supervision data. As detailed in Table 2, these features are explicitly selected to represent the physical, geographical, and topographical distortions that typically induce radar and optical sensor errors.

To eliminate dimensional dominance and accelerate model convergence, all continuous features were standardized to zero mean and unit variance (Z-score normalization) prior to model ingestion.

### 3.2. Model Architecture and Implementation

To rigorously test the “Physics versus Geometry” hypothesis and establish the boundaries of different modeling paradigms, we constructed a comparative framework encompassing three distinct approaches: (1) Classical Geospatial Interpolation, utilizing pure spatial autocorrelation; (2) Explicit Feature-Based Learning, relying on deterministic physical variables; and (3) Implicit Spatial Representation Learning, leveraging geometric deep learning. Furthermore, we designed a Hybrid Architecture to investigate the potential synergy between physical and geometric features.

#### 3.2.1. Classical Geospatial Interpolation Baselines

To benchmark the machine learning approaches against traditional geostatistics, we selected two widely established methods that rely solely on the spatial distribution of errors.

(1) Inverse Distance Weighting (IDW) [64] adheres strictly to Tobler’s First Law of Geography, performing a weighted average of k surrounding known points, where the weight ($w_{i}$) is inversely proportional to the geographic distance.

(2) $$w_{i}=\frac{1}{d_{i}^{p}}$$

where $d_{i}$ is the distance from the i-th neighboring point to the prediction point, and p is the power parameter that controls the rate of weight decay.

The number of neighbors (k) and the power parameter (p) were optimized via grid search to achieve the best performance.

(2) Ordinary Kriging (OK) [65,66] provides the Best Linear Unbiased Prediction (BLUP) by explicitly modeling the spatial autocorrelation structure. To mitigate the $O(N^{3})$ computational complexity associated with large-scale datasets, we implemented a sliding-window Local Ordinary Kriging approach, automatically selecting theoretical variogram models based on minimal validation RMSE within localized k-d tree (k = 25) neighborhoods.

#### 3.2.2. Explicit Feature-Based Learning

This paradigm assumes that DEM error is a deterministic function of local surface characteristics (defined in Section 3.1) and treats each sample as an independent entity in the decision space.

(1) XGBoost (eXtreme Gradient Boosting) [20] was selected as the representative state-of-the-art model for tabular regression. Its ensemble learning strategy, which iteratively constructs a series of decision trees fitted to residuals, renders it exceptionally effective at capturing complex, high-order non-linear relationships among physical features.
(2) Multilayer Perceptron (MLP) [67] serves as a standard deep learning baseline for non-graph-structured data, enabling an effective ablation study to evaluate the performance gain strictly attributable to the introduction of graph structures in subsequent GNN models.

#### 3.2.3. Implicit Spatial Representation Learning

Unlike Euclidean grids, the Lagrangian nature of ICESat-2 orbital tracks renders conventional Convolutional Neural Networks (CNNs) ill-suited. In contrast, Graph Neural Networks (GNNs) operate on non-Euclidean domains, making them mathematically ideal for modeling discrete topological dependencies [29]. We induced a synthetic local topology using the k-Nearest Neighbor (k-NN) algorithm [68], establishing edges based on Euclidean distance to transform independent error points into a connected system G=(V,E). For each node $v_{i}$, we established edges to its k = 8 spatially nearest neighbors based on Euclidean distance.

**Figure 4:**
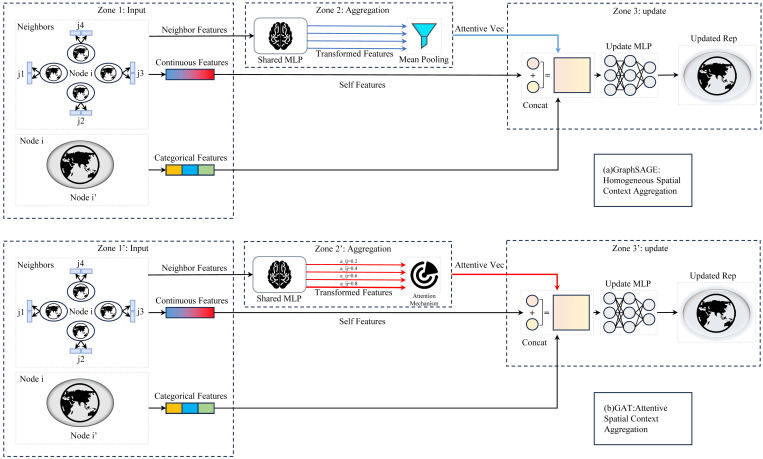
Schematic of GNN message passing. (**a**) GraphSAGE. (**b**) GAT. The blue and red arrows distinguish the distinct aggregation and propagation paths for GraphSAGE and GAT, respectively.

(1) GraphSAGE [35] was employed to model “homogeneous” spatial dependencies via isotropic aggregation, acting as a localized smoothing filter by mean-pooling sampled neighborhood features and testing the hypothesis that DEM errors are spatially continuous and locally consistent.
(2) Graph Attention Network (GAT) [36] overcomes isotropic limitations by learning dynamic ‘attention weights’ based on central-neighbor node similarity. This anisotropic message-passing captures complex spatial dependencies more granularly. Furthermore, we incorporated the inverse geographic distance between nodes as an edge attribute within the GATv2Conv layer, making the attention score sensitive to both node features and geographic proximity (Figure 4).

#### 3.2.4. The Hybrid “Dual-Drive” Architecture

**Figure 5:**
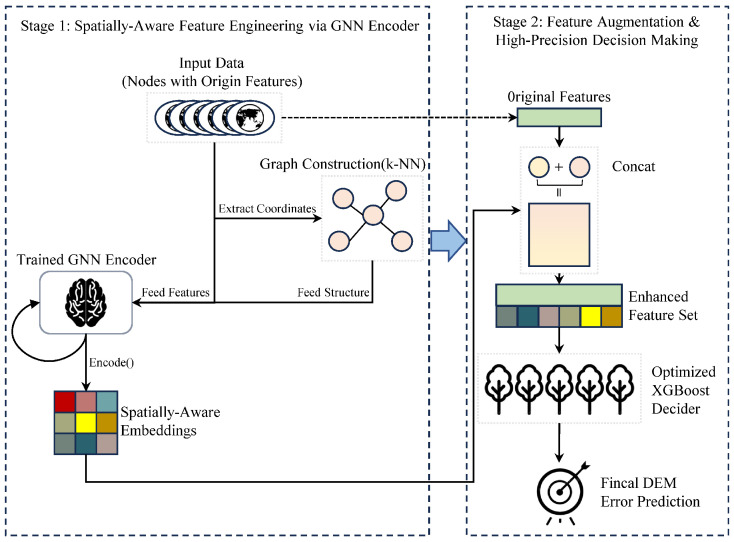
Architecture of the hybrid GNN-XGBoost model. Solid arrows indicate the primary data processing workflow, while the dashed arrow represents the direct passing of original input features to the concatenation stage. In the feature representations, the uniform green blocks denote the original explicit physical features, whereas the multi-colored grid blocks conceptually illustrate the high-dimensional, latent spatially-aware embeddings generated by the GNN.

To comprehensively evaluate whether “Implicit Geometry” can complement “Explicit Physics,” we designed a decoupled GNN-XGBoost Hybrid Model. The architecture of this model is illustrated in Figure 5, operating through a three-stage workflow: (1) GNN Encoder Training: A GraphSAGE model is trained independently to directly predict DEM errors. (2) Spatial Feature Extraction: The trained GNN encoder extracts the output of its penultimate layer for each node, yielding a 128-dimensional ‘spatially aware embedding’ that encapsulates fused neighborhood context. (3) Concatenation and Decision Making: We concatenate this 128-dimensional spatial embedding with the original physical feature vector to form an augmented feature set, which is then ingested by an optimized XGBoost model.

Regarding the fusion of these disparate feature sets, we deliberately eschewed a gradient-based learnable joint-gating mechanism in favor of a Concatenation-to-Boosting strategy. Explicit physical variables (e.g., slope, aspect) and latent topological embeddings occupy vastly distinct numerical distributions and semantic spaces. Attempting end-to-end joint optimization with a parametric gate on such hybrid tabular/embedding structures frequently precipitates gradient scaling imbalances and severe overfitting. Conversely, by concatenating the features and employing XGBoost as the final decision-maker, we exploit the intrinsic information-gain-based tree-splitting mechanism of gradient boosting. The decision trees inherently act as robust, non-linear, and dynamic gates—automatically evaluating, selecting, and fusing the most discriminative variables from both physical and spatial domains without introducing the parametric instability of an auxiliary neural gating layer.

This hybrid model is designed to test a core hypothesis: whether the spatial context automatically learned by a GNN can serve as an effective, automated feature engineering tool to provide a performance boost to a powerful conventional model.

### 3.3. Experimental Design

To ensure a rigorous and equitable comparison between different modeling paradigms, we implemented a standardized experimental protocol covering data partitioning, optimization strategy, and hyperparameter configuration.

#### 3.3.1. Data Partitioning and Seed Control

The dataset for each DEM was first partitioned into a development set (80%) and a hold-out test set (20%) using a fixed random seed (random_state = 42) to ensure absolute reproducibility. The development set was further subdivided into training and validation subsets (80/20 split) for Bayesian hyperparameter optimization. For deep learning models (MLP, GraphSAGE, GAT, and Hybrid), a 15% validation subset was held out from the development set specifically for early stopping to prevent overfitting during the final training stage.

#### 3.3.2. Automated Hyperparameter Optimization

To mitigate manual tuning bias, model performance was optimized using the Optuna framework [69], which utilizes Bayesian optimization to navigate pre-defined search spaces. Each learning-based model underwent 50 optimization trials (n_trials = 50) to minimize the Root Mean Square Error (RMSE) on the validation subset. For the traditional IDW, a systematic grid search was performed, while for Ordinary Kriging (OK), an automated least-squares variogram fitting procedure was implemented to ensure the spatial structure was optimally captured.

#### 3.3.3. Implementation and Parameter Settings

The experimental framework was implemented in Python, utilizing XGBoost (v3.1.2) for gradient boosting, PyTorch (v2.0.1) and PyTorch Geometric (v2.6.1) for neural networks, and PyKrige (v1.7.3) for geostatistical modeling. For graph-based models, spatial weight graphs were constructed using a K-Nearest Neighbors (KNN) approach with k = 8, a value determined based on the average density of ICESat-2 footprints. The detailed search spaces and optimization settings for all models are summarized in Table 3.

### 3.4. Evaluation Metrics and Explainability Analysis Methods

#### 3.4.1. Reference Data Quality Control

To ensure the reliability of the reference data and minimize the impact of gross errors (e.g., cloud contamination), a statistical filtering approach was applied. For each control point, the elevation discrepancy was calculated as $dH=H_{ATL08}-H_{DEM}$. Assuming random errors follow a normal distribution, we applied the 3σ rule [70]. Points with dH falling outside the range [μ−3σ,μ+3σ] (where μ and σ denote the mean and standard deviation) were identified as outliers and excluded from the dataset.

#### 3.4.2. Quantitative Accuracy Metrics

The quantitative evaluation of point-wise DEM accuracy relies on four statistical metrics: Root Mean Square Error (RMSE), Mean Absolute Error (MAE), Mean Error (ME), and the outlier-robust Normalized Median Absolute Deviation (NMAD) [71,72,73].

(3) RMSE=1n∑i=1n(xi−x^i)2,

(4) $$NMAD=1.4826\times \mathrm{median}(|x_{i}-\mathrm{median}(x)|),$$

(5) $$\mathrm{MAE}=\frac{1}{n}\sum_{i=1}^{n}|x_{i}-x_{i}|,$$

(6) $$ME=\frac{1}{n}\sum_{i=1}^{n}\left(x_{i}-x_{i}\right),$$

where $x_{i}$ and x^i represent the evaluated DEM and reference ATL08 elevations, respectively, and median (x) denotes the median of the evaluated dataset.

#### 3.4.3. Explainability and Geomorphometric Fidelity

Beyond point-wise accuracy, we employed explainable AI techniques to compare the internal decision-making mechanisms between “Explicit Physical Features” (local attributes like slope/aspect acting as direct radar distortion proxies) and “Implicit Spatial Relations” (latent topological dependencies dynamically learned by GNNs).

1. SHAP (SHapley Additive exPlanations) [37]: We utilized the TreeExplainer algorithm to quantify the marginal contribution (Shapley value) of explicit features in XGBoost. Furthermore, for the GNN-XGBoost hybrid, SHAP allows for a quantitative assessment of the overall predictive value of the GNN-generated spatial embeddings.
2. GNNExplainer [38]: Applied to the GraphSAGE and GAT models, this method extracts the most influential computational subgraphs via mutual information maximization. By averaging the feature importance masks across sample nodes, we derive a global ranking to compare against the SHAP analysis.
3. Geomorphometric Fidelity Assessment: To evaluate terrain structure preservation without introducing rasterization interpolation errors, we introduced the Slope Fidelity metric. The instantaneous Along-Track Slope ($S_{AT}$) was computed directly from vector-based ICESat-2 photon footprints:
  (7) $$S_{AT}=\arctan\left(\frac{H_{i+1}-H_{i}}{D_{i,i+1}}\right)\times \frac{180}{\pi },$$
  where $D_{i,i+1}$ is the geodesic distance. To capture dominant topographic gradients while filtering data gaps, computations were restricted to a meso-scale threshold (0.5 m < D < 300 m). The structural fidelity was then quantified using the RMSE of the model-derived $S_{AT}$ against the reference.

## 4. Results

### 4.1. The Performance Hierarchy

**Figure 6:**
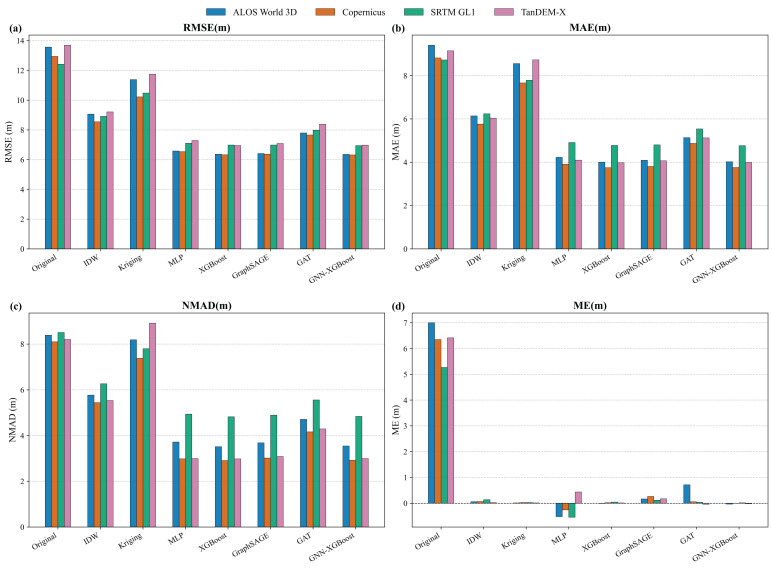
Bar chart comparing model performance across four DEM datasets. (**a**) RMSE, (**b**) MAE, (**c**) NMAD, and (**d**) ME.

As illustrated in Table 4 and Figure 6, a stable performance hierarchy consistently emerges across all four DEM datasets: Hybrid ≈ XGBoost > GraphSAGE > MLP > GAT > IDW > Kriging. This clear stratification validates our core hypothesis regarding feature utility:

(1) The Robustness of Physical Features against Geometric Over-Complexity (MLP vs. GAT): As shown in Table 4, while GraphSAGE holds a slight edge over MLP, the attention-based GAT performs significantly worse than both, occupying a lower tier (e.g., RMSE 6.581 m for MLP vs. 7.793 m for GAT on AW3D). This directly explains why overly complex geometric approaches struggle in this specific task. In sparse, high-relief reference scenarios, explicit physical features (e.g., aspect) strictly govern and correct the dominant radar/optical distortions with high robustness. Conversely, as geometric models increase in complexity, they suffer a severe “complexity penalty.” GAT faces attention saturation and overfits to the sparse, irregular spatial topologies, failing to extract reliable implicit spatial relationships. Thus, a robust physical baseline (MLP) naturally surpasses a brittle, overly complex spatial model (GAT), further proving that established physical laws are far more reliable than forced geometric inferences in data-sparse environments.

(2) The Failure of Pure Spatial Autocorrelation (Kriging & IDW): Geostatistical baselines occupy the bottom tier (RMSE > 8 m), confirming that DEM errors in complex topography are deterministic and physically driven, not merely a function of spatial proximity.

#### Diminishing Returns and the Complexity Trade-Off

While the Hybrid model consistently achieves the lowest absolute RMSE across datasets (e.g., improving upon XGBoost by a marginal 0.013 m on AW3D), the improvement is remarkably marginal (<0.05 m). So, we must evaluate whether this gain justifies the computational overhead. To strictly assess this “RMSE parity,” we conducted a Wilcoxon signed-rank test between the Hybrid (k = 8) and XGBoost results. Despite the small absolute margin, the differences are statistically significant (Bonferroni-corrected *p* < 0.001). However, assessing this statistical improvement through a Performance vs. Complexity lens reveals a stark trade-off. As detailed in the computational cost analysis (tested on identical hardware), the pure XGBoost model achieves near-optimal correction utilizing only CPU resources with a rapid training time (~36.86 s). In contrast, introducing the GNN encoder necessitates GPU allocation and increases the pre-training time to roughly 666.23 s—a nearly 18-fold increase in computational cost for an RMSE reduction of less than 0.7%. This massive computational asymmetry, combined with marginal accuracy gains, compellingly demonstrates that explicit physical features (the “Physics Skeleton”) already explain the vast majority of the error variance. The implicit spatial embeddings provide merely a negligible supplementary effect, solidifying the operational superiority of the purely physical XGBoost model for large-scale DEM correction tasks, see Table 5.

### 4.2. Beyond Point Precision: Geomorphometric Fidelity Evaluation

Beyond vertical point accuracy, a critical objective is determining if the model genuinely restores the structural fidelity of the terrain rather than merely removing a systematic global bias. To evaluate the correction of high-frequency topographic errors, we implemented a Lagrangian Along-Track Approach. This method mitigates the spatial support inconsistency between discrete laser footprints and continuous raster grids. Specifically, we calculated the Instantaneous Along-Track Slope ($S_{AT}$) directly between adjacent ICESat-2 footprints and compared it with the slope between the corresponding coordinates on the DEM surfaces. This guarantees an identical spatial baseline for a strict terrain structure comparison.

**Figure 7:**
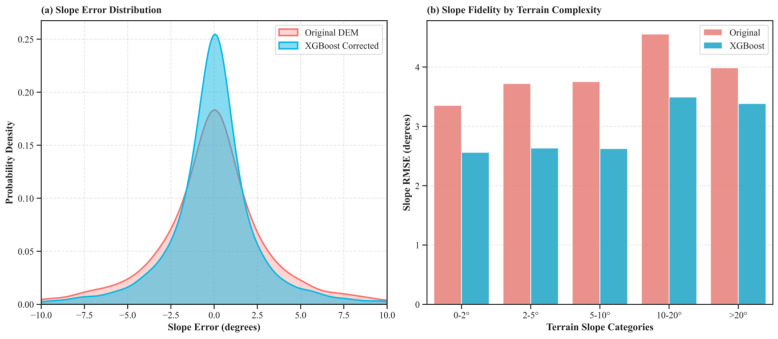
Assessment of geomorphometric fidelity using the AW3D dataset via the along-track slope comparison method. (**a**) Probability density estimation (KDE) of the slope errors. The XGBoost-corrected distribution (blue) shows a sharper peak and narrower tails compared to the original DEM (red), confirming the reduction in random structural noise. (**b**) Slope RMSE stratified by terrain slope categories. The correction reduces gradient errors across all terrain complexities, reducing the overall Slope RMSE from 3.97° to 3.06°, demonstrating that the model preserves topographic realism better than the raw data.

Evaluating 7007 valid slope pairs from the AW3D dataset (selected for its high sensitivity to roughness, detailed in Section 4.5), the results provide compelling evidence of geomorphometric restoration (Figure 7).

The Kernel Density Estimation (KDE) in Figure 7a illustrates that the original DEM’s slope error distribution exhibits heavy tails. Conversely, the XGBoost-corrected distribution is significantly sharper and centered near zero. Quantitatively, the overall Slope RMSE decreased from 3.97° to 3.06° (a 23.07% relative improvement). This substantial reduction confirms that the model effectively corrects non-stationary, spatially varying errors rather than applying a simple mean-shift.

While traditional smoothing models typically fail in high-gradient areas by underestimating peaks and valleys, Figure 7b demonstrates pervasive correction across all terrain complexities. The explicit feature-based model maintains highly robust performance even in the most challenging topography (Slope > 20°).

These findings firmly reinforce our core objective and hypothesis: explicit physical learning dynamically adjusts the correction magnitude based on local morphometric contexts (e.g., Slope). Rather than suffering from the low-pass filtering effect inherent to implicit spatial interpolation, the XGBoost model acts as a “texture synthesizer,” successfully re-sculpting distorted landforms and preserving topographic realism.

### 4.3. Decision Mechanisms: A Tale of Three “Worldviews”

**Figure 8:**
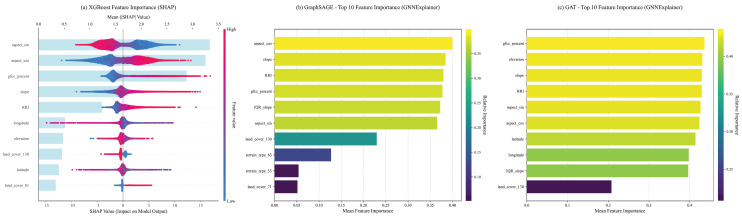
A comparison of the decision-making ‘worldviews’ of the three models. (**a**) SHAP feature importance for XGBoost. The light blue bars represent the mean absolute SHAP value, indicating the overall importance of each feature; (**b**) GNNExplainer feature importance for GraphSAGE; (**c**) GNNExplainer feature importance for GAT.

To investigate the underlying causes of the performance disparities described in Section 4.1, we conducted an in-depth explainability analysis using SHAP (for XGBoost) and GNNExplainer (for GraphSAGE and GAT). Selecting the CopDEM dataset as a representative case, the results (Figure 8) reveal that despite receiving an identical set of input features, the models exhibit three fundamentally different decision-making “worldviews” that dictate their performance.

(a) The “Discriminative Physicist” Worldview of XGBoost

XGBoost exhibits a highly discriminative and sparse decision logic, aggressively isolating the dominant error drivers (Figure 8a). It strictly prioritizes sensor geometry, with the trigonometric components of aspect (aspect_sin, aspect_cos) emerging as the paramount determinants (mean SHAP ~3.3). Vegetation cover (gfcc_percent) follows as the secondary tier, while local morphometric factors (slope, RRI) are assigned lower supplementary weights. This rigid hierarchy (Sensor Geometry > Surface Medium > Local Texture) aligns perfectly with the physical principles of remote sensing distortions.

(b) The “Isotropic Mean Aggregator” Worldview of GraphSAGE

Conversely, GraphSAGE demonstrates a “flattened” perception of physical reality (Figure 8b). The importance scores of its top features (aspect_cos, slope, RRI, gfcc_percent) are tightly clustered within a negligible margin (0.37–0.40). By averaging feature information across local neighborhoods, its isotropic aggregation mechanism effectively “washes out” the distinct, high-frequency signals of specific physical drivers. It creates a generalized “feature soup” where no single physical law dominates.

(c) The “Attention Homogenization” Worldview of GAT

The GAT model suffers from profound attention saturation (Figure 8c). Its importance scores are even more compressed than GraphSAGE’s, with the top five features all falling within a narrow range of 0.42–0.44. Struggling with feature collinearity, GAT attempts to leverage all correlated physical signals simultaneously with equal weight. This indecisiveness introduces information redundancy and prevents the model from locking onto the primary error source.

By systematically comparing these mechanisms, it becomes clear why explicit physical learning outperforms implicit geometry in this context: XGBoost accurately isolates physical causality, whereas complex GNNs suffer from feature homogenization when applied to sparse, high-relief geomorphometric data.

### 4.4. Stable Performance, Drifting Explanations

As established in Section Diminishing Returns and the Complexity Trade-Off, the inherent stochasticity in training the GNN-XGBoost Hybrid model does not affect its macroscopic predictive accuracy; the RMSE remains highly stable across independent runs (Table 6). However, an in-depth analysis of the model’s internal decision logic reveals a significant methodological phenomenon, where the model achieves consistent predictions despite relying on highly variable, drifting feature representations.

To rigorously investigate this, we conducted three independent training sessions using both the pure XGBoost and Hybrid models. We then evaluated the feature attribution consistency globally across the dataset and locally within a specific high-relief geographic feature (a high mountain sub-region from Figure 2a).

**Figure 9:**
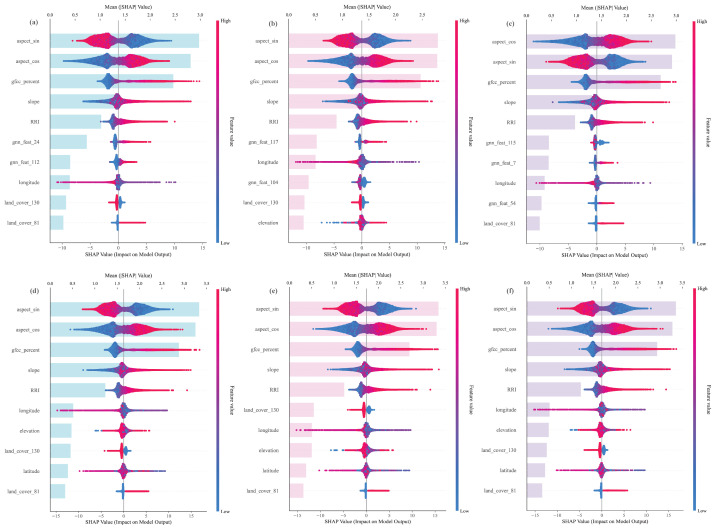
SHAP Top-10 feature importance plots for the XGBoost and Hybrid model on the CopDEM dataset across three independent runs. The first row (**a**–**c**) represents the SHAP results of the hybrid model, while the second row (**d**–**f**) represents the SHAP results of the XGBoost model.

At the global scale, while explicit physical features (Aspect, Vegetation) remained consistently ranked at the top, the ranking of GNN-generated latent features fluctuated wildly across runs for the Hybrid model (Figure 9). Pairwise rank correlation (ρ) analysis [74] across experiments (Table 7) confirmed that the pure XGBoost model achieved near-perfect stability (ρ > 0.97), whereas the Hybrid model exhibited significant inconsistency (ρ ≈ 0.63–0.77).

**Figure 10:**
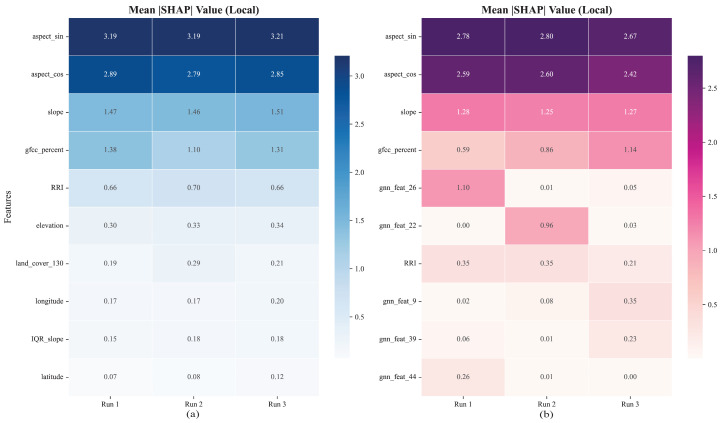
Visualization of local attribution drift in a high-mountain region. Heatmaps display the Local Mean |SHAP| values over three independent training runs for (**a**) the pure XGBoost model, demonstrating highly consistent physical feature attribution, and (**b**) the Hybrid model, illustrating significant attribution drift among implicit spatial features.

To illustrate the geographic implications of this drifting explanation, we mapped the local mean SHAP values for the high-mountain test region using a heatmap (Figure 10).

The pure XGBoost model demonstrates absolute determinism (Figure 10a). Across all three runs, the primary physical drivers—such as aspect_sin (~3.20), aspect_cos (~2.84), and slope (~1.48)—maintain nearly identical SHAP magnitudes. The model consistently recognizes the exact physical causes of elevation error.

In stark contrast, the Hybrid model exhibits severe local attribution drift (Figure 10b). While the overarching physical features remain present, the implicit spatial embeddings “take turns” explaining the local residuals. In Run 1, the model heavily relies on gnn_feat_26 (1.10); in Run 2, this feature drops to 0.01, and gnn_feat_22 emerges (0.96); in Run 3, entirely different features (gnn_feat_9) take over.

This drift is not a computational error but a fundamental characteristic of applying over-parameterized geometric models to sparse geomorphometric data. The GNN possesses excessive degrees of freedom, allowing it to find multiple, equally valid mathematical representations of the same local spatial noise. Therefore, while the Hybrid model can marginally improve accuracy by opportunistically fitting local residuals, its explanatory logic is mathematically non-unique. The resolution to this methodological instability is to rely on the explicitly constrained physical feature space (XGBoost), which guarantees both high accuracy and deterministic, physically interpretable causality.

### 4.5. The Opportunism of GNN Features

**Figure 11:**
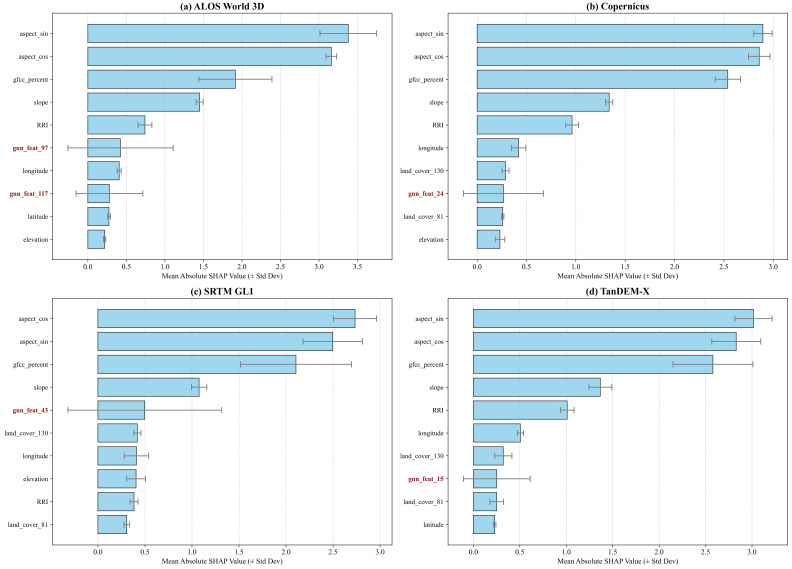
Average Top-10 Feature Importance of the Hybrid Model Across DEMs. (**a**) AW3D. (**b**) CopDEM. (**c**) SRTM. (**d**) TanDEM. The feature names highlighted in red on the y-axis indicate the spatially-aware embeddings generated by the GNN encoder.

Having established the stochasticity of the Hybrid model’s feature attribution within a single dataset, we further analyzed the average SHAP feature importance across all four DEM products (Figure 11) to determine if the extracted implicit spatial relations represent universal physical laws or merely dataset-specific adaptations. The systematic cross-dataset comparison reveals a clear behavioral dichotomy between explicit and implicit features.

The Universality of Explicit Physics: Across all four fundamentally different DEMs, explicit physical variables—specifically Acquisition Geometry (aspect_sin, aspect_cos) and Forest Cover (gfcc_percent)—rigidly dominate the Top-3 positions. This absolute consistency proves that the primary systematic elevation errors (e.g., radar foreshortening and canopy penetration) are universally governed by these explicit geomorphometric descriptors. They function as a highly robust and transferable error correction model.

The Dataset-Dependent Opportunism of GNN Features: In stark contrast, the contribution of GNN-derived implicit spatial features (gnn_feat_xx) exhibits significant instability and opportunistic behavior. First, their specific latent identities vary randomly across datasets (e.g., relying on gnn_feat_97 for AW3D versus gnn_feat_43 for SRTM), lacking any universal spatial motif. Second, their importance strictly scales with the dataset’s inherent noise level. For instance, a GNN feature rises to Rank 5 in the noisier SRTM dataset, but drops below Rank 8 in high-precision products like CopDEM.

This systematic evaluation firmly concludes that the GNN component does not learn a universal terrain correction logic; rather, it acts as a dataset-dependent “local residual scavenger.” It merely identifies specific latent vectors that opportunistically correlate with the remaining noise in the training data. Consequently, while implicit spatial relations can offer marginal local improvements, the explicitly engineered physical features unequivocally form the indispensable, generalizable “skeleton” of the correction model.

## 5. Discussion

### 5.1. Physics Trumps Geometry

While geometric deep learning thrives in dense spatial modeling, our results (Section 4.1) reveal diminishing returns when applying it to pixel-based geomorphometric error correction under sparse supervision. Consistent with recent machine learning literature—which notes that deep learning architectures do not inherently outclass gradient-boosted decision trees on tabular data structures [75]—our findings demonstrate that the Hybrid model’s marginal accuracy gains (ΔRMSE < 0.05 m) are eclipsed by a nearly 18-fold increase in computational cost (Table 5). This statistical parity, when contrasted with their fundamentally disparate decision mechanisms (Section 4.3), exposes a fundamental dichotomy: the deterministic reliability of physical laws versus the stochastic nature of over-parameterized geometric learning.

#### 5.1.1. Explicit Features as the Deterministic Skeleton

The robust performance of XGBoost stems from its “Discriminative Physicist” worldview (Figure 8a), which strictly mirrors the physical mechanisms of DEM error generation. The dominance of specific explicit features is not a statistical artifact but a reflection of physical causality:

Acquisition Geometry: The absolute dominance of aspect_sin and aspect_cos (mean SHAP ~3.3) physically maps to the systematic side-looking biases inherent in InSAR sensors (e.g., foreshortening and layover) and solar illumination shadows in optical stereo-photogrammetry.

Penetration Depth: The secondary importance of gfcc_percent explicitly accounts for the canopy penetration bias, effectively isolating the bare-earth signal from the surface medium.

This explicit physical skeleton is indispensable in complex topography. While purely spatial interpolation (e.g., Kriging, IDW) might demonstrate acceptable generalization in flat plains where geometric distortions are minimal, high-relief areas demand rigorous geophysical constraints to prevent severe structural underestimations [76,77,78]. By accurately mapping these high-signal-to-noise inputs, XGBoost validates the efficacy of machine learning as a framework for exploring physical geographic relationships [79,80].

#### 5.1.2. The Decoupling Hypothesis: Right for the Wrong Reasons

In stark contrast, the GNN-derived implicit spatial features exhibit a dangerous decoupling between macroscopic accuracy and microscopic causality—a classic manifestation of being “right for the wrong reasons” (fitting spatial noise rather than learning geomorphology). We substantiate this “Decoupling Hypothesis” with three empirical findings from Section 4.4 and Section 4.5:

Attribution Instability: While the Hybrid model maintains macroscopic predictive stability, its internal reliance on specific features is highly volatile across independent runs (ρ≈ 0.63–0.77), significantly lagging behind the crystallized logic of XGBoost (ρ > 0.97) (Table 7).

ID Chaos: The specific latent dimensions identified as “important” fluctuate randomly across datasets (Figure 11) and even within the same local high-mountain region (Figure 10b). GNNs fail to establish a universal topological motif.

Noise Dependency: The relative importance of GNN features strictly scales with the inherent noise level of the dataset (e.g., ranking higher in the noisy SRTM than in the high-precision CopDEM), proving they act as opportunistic “local residual scavengers” rather than discoverers of physical laws.

Consequently, in tasks endowed with strong physical signals, the interpretability and structural robustness of a “Physics-First” model (XGBoost) unequivocally outweigh the marginal, indeterminate gains of a complex geometric “black box.” This conclusion strongly resonates with the broader call for interpretable AI in high-stakes scientific decision-making [81,82].

### 5.2. Scale Mismatch in Graph-Based Inference

While the core findings highlight the robust performance of explicit feature engineering, the relative limitations of GNNs provide invaluable insights for defining the applicability boundaries of geometric deep learning in geomorphometry. The performance bottleneck of the Hybrid model is not merely a structural deficiency, but rather rooted in a fundamental scale mismatch between the model’s inductive bias, the multi-scalar nature of DEM error, and the severe sparsity of the supervisory data.

#### 5.2.1. Scale Mismatch: Point-Wise vs. Graph-Based Inference

A critical constraint in global DEM correction is the spatial support of the reference data. The ICESat-2 altimetry data provides high-precision but spatially discrete footprints, introducing a severe scale mismatch for graph-based models. GNNs are fundamentally designed under the assumption of a dense, connected topology to perform effective message passing [83].

To mathematically contextualize this paradox—and directly address the physical viability of graph construction in this context—we quantified the spatial distribution of the 363,934 valid ICESat-2 reference footprints within our study area. When constructing the graph topology (k = 8), the average physical distance between connected nodes reaches 485.25 m, with a median distance of 401.72 m. Furthermore, in areas of extreme data sparsity, the maximum cross-track linkage stretches up to an astonishing 32,726.75 m.

This massive spatial baseline fundamentally violates the dense, localized neighborhood assumption inherent to standard GNNs. Consequently, the message-passing mechanism acts as a detrimental smoothing filter; by forcing linkages across vast geographical distances, it mixes highly heterogeneous terrain features and induces a severe “over-smoothing” phenomenon where high-frequency topographic details are irreversibly lost [83].

In stark contrast, XGBoost treats each sample independently as a point-wise regressor. It is completely agnostic to the spatial density of the training set, allowing it to robustly map isolated physical features to error values without requiring an artificial and heavily distorted neighborhood structure.

#### 5.2.2. The Receptive Field Mismatch

Beyond data sparsity, there is an intrinsic conflict between the local receptive field of GNNs and the multi-scalar nature of systematic DEM errors, which encompass both local random noise and pervasive low-frequency biases caused by orbital maneuvers, sensor jitter, and atmospheric path delays [84]. Our explainability analysis (Section 4.3) provides compelling evidence for this scale mismatch:

Global Awareness (XGBoost): As demonstrated in the SHAP analysis (Figure 8a), the global coordinate features (Latitude and Longitude) consistently rank high in importance alongside local terrain factors. This indicates that XGBoost successfully approximates a global trend surface to correct large-scale absolute systematic errors.

Local Myopia (GNNs): Conversely, standard GNN architectures are permutation-invariant and operate under a strict locality assumption. Without explicit and sophisticated positional encoding, a GNN node is blind to its absolute position within the continental-scale study area [85]. This local myopia forces the GNN to erroneously attempt to fit global systematic biases using only fragmented local neighborhood features, further explaining the “ID Chaos” observed in Section 5.1.

This structural limitation tightly links our research to the classic problem of scale in geography [86], proving that for geospatial error correction tasks—where the signal is global but the reference data is sparse—simple independent models like XGBoost are structurally far more robust than graph models demanding dense, coherent topologies.

### 5.3. Underspecification and the Challenge of Explanatory Stability

This study, through rigorous repeated experimentation, unveils a phenomenon of critical importance to the emerging field of Geospatial Explainable AI (Geo-XAI) [87]: “stable performance, drifting explanation.” While previous sections established the physical superiority of explicit features, the stochastic behavior of the Hybrid model exposes a foundational crisis in trust.

#### 5.3.1. The Phenomenon of Underspecification

As quantitatively demonstrated in Section 4.4, there is a disturbing divergence between predictive reproducibility and attribution stochasticity.

Predictive Reproducibility: The Hybrid model maintains high stability in macroscopic metrics (e.g., RMSE, MAE) across independent runs (Table 6).

Attribution Stochasticity: Conversely, the SHAP rankings exhibit severe volatility. The Hybrid model lacks the crystallized logic observed in XGBoost, with its Spearman rank correlation (ρ) dropping to approximately 0.63–0.77 compared to XGBoost’s near-perfect stability (ρ > 0.97) (Table 7).

This disconnect is a textbook manifestation of “Underspecification” [88]. In high-dimensional geometric optimization on sparse datasets, the training pipeline is heavily underspecified. The GNN essentially “guesses” a mathematically valid but physically disparate path to the same error minimum in each run, proving that its reliance on implicit spatial features is a probabilistic artifact rather than a discovered scientific law.

#### 5.3.2. The Fragility of “Black Box” Insights

The profound implication of this finding is that XAI results from a single deep learning training run must be treated with extreme skepticism.

A common pitfall in current Geo-AI research is to equate model saliency (feature importance) directly with real-world physical causality. However, our findings echo foundational critiques in machine learning, which warn that neural network interpretations are often fragile—sensitive to minor perturbations like random seed initialization or data loading order [89]—and may fail basic sanity checks for reliability [90].

Our local attribution analysis (Figure 10) vividly illustrates this fragility. In the high-mountain test region, the Hybrid model’s explanation for local residuals completely shifted across three identical runs: prioritizing gnn_feat_26 initially, then shifting to gnn_feat_22, and finally relying on an entirely different gnn_feat_9. Drawing geomorphometric conclusions based on a random snapshot of this stochastic process would inevitably lead to scientifically invalid narratives.

In stark contrast, constrained by the “Physics Skeleton” of explicit features (Aspect, Vegetation), the pure XGBoost model consistently isolates identical physical causes (ρ > 0.97), proving that true explanatory stability is a direct function of rigorous physical constraints.

#### 5.3.3. The Imperative for Stability Metrics

Consequently, we argue that the Geo-AI community must evolve beyond the singular pursuit of accuracy (e.g., RMSE/MAE). Future studies aiming to derive scientific insights from model explanations have a responsibility to establish explanatory stability as a core evaluation metric.

Researchers should quantitatively assess the robustness of their explanations through multiple repeated experiments, reporting metrics such as the coefficient of variation or Spearman’s rank correlation of feature importance. Only features that consistently emerge as dominant across multiple runs—like the Aspect and Vegetation features in our XGBoost model—can be trusted as reflecting true, underlying geographic processes. Accuracy is a measure of utility; stability is the measure of scientific truth.

### 5.4. Implications, Limitations, and Future Roadmap

While this study has delineated the dominance of explicit physical features through comprehensive experimentation, it is imperative to prudently acknowledge the boundary conditions of these findings and chart a rigorous course for future Geo-AI research.

#### 5.4.1. Boundary Conditions and Limitations

The validity of our “Physics Trumps Geometry” conclusion is subject to specific structural and geographic constraints:

Geographic Generalizability: Our study area (Sichuan) represents a “natural laboratory” of extreme topographic complexity. While this validates the robustness of the “Physics Skeleton” in challenging terrains, the behavior of implicit spatial features in physically homogeneous environments (e.g., vast plains or ice sheets) remains to be verified.

The Static Graph Constraint: As elucidated in Section 5.2, our GNN models relied on static K-NN graphs constructed from sparse ICESat-2 footprints. Forcing this rigid static topology across massive physical distances (up to ~32 km) fails to capture dynamic, anisotropic error correlations that vary with terrain aspect or sensor flight direction.

Systemic Noise Floor: The conversion to ellipsoidal heights utilized the EGM2008 geoid model, introducing an intrinsic global uncertainty of approximately ±0.5–1.5 m. According to the principles of error propagation, this systemic uncertainty acts as an unresolvable noise floor. Crucially, this noise affects all evaluated models uniformly; therefore, it preserves the relative performance hierarchy between the feature-engineered (XGBoost) and geometric (GNN) architectures, solidifying our core thesis.

Architectural Scope: We focused on canonical architectures (GraphSAGE, GAT) to isolate the fundamental mechanism of spatial induction. We did not exhaustively explore cutting-edge variants like dynamic graph evolution, although our results suggest that without addressing the underlying data sparsity, architectural complexity alone yields diminishing returns.

#### 5.4.2. Future Roadmap: A Call to the Geo-AI Community

(1) Redefining GNNs for Feature-Sparse Regimes

Our findings suggest that in “feature-rich” tasks (where explicit physical gradients like Aspect and Vegetation are available), GNNs act primarily as residual scavengers. Therefore, the true potential of GNNs may lie in “feature-sparse” contexts. Future studies should investigate scenarios where high-quality auxiliary data is unavailable (e.g., planetary mapping or bathymetry). In such regimes, GNNs might transition from “residual-dependent latent feature learners” to “essential reconstructors,” learning effective representations purely from latent geospatial relationships to surpass traditional interpolation methods [91].

(2) Advancing towards Dynamic Graphs and Global Attention

To resolve the “Local Myopia” and the static K-NN bottlenecks identified in our sparse data regime, future architectures must integrate dynamic multi-scale awareness. A critical specific direction is the application of Graph Transformers and Dynamic Edge Convolution paradigms. These dynamic graphs can actively reconstruct topologies based on latent feature similarity rather than rigid physical distance, while self-attention mechanisms allow the model to simultaneously attend to local terrain roughness and global orbital trends [92]. This aligns seamlessly with emerging paradigms for processing non-Euclidean geospatial data [93].

(3) Institutionalizing Explanatory Stability

We issue a strong call to the Geo-AI community to move beyond the singular pursuit of predictive accuracy (RMSE). The “Attribution Stochasticity” revealed in this study underscores a crisis of trust in spatial deep learning. Future research must embrace “Neuro-symbolic” or “Grey Box” approaches that enforce hard physical constraints within the loss function (e.g., Physics-Informed Neural Networks) or co-optimize deep learning representations with gradient-boosted decision trees [94]. Furthermore, we advocate for the adoption of Stability Metrics (e.g., attribution rank correlation) as a standard publication requirement. Only by ensuring model explanations are mathematically deterministic can we rely on AI for genuine scientific discovery.

## 6. Conclusions

To address the unresolved question regarding the superiority of the two prevailing paradigms in DEM error correction—explicit feature engineering versus implicit geometric deep learning—this study executed a rigorous comparative framework across geomorphologically diverse landscapes. By integrating high-precision ICESat-2 altimetry data with explainable machine learning, we elucidate the mechanistic boundaries of these models and arrive at the following core conclusions:

(1) First, Physics Trumps Geometry in Sparse Data Regimes.

Contrary to the prevailing assumption that graph-based architectures superiorly capture terrain topology, our results demonstrate that the GNN-XGBoost Hybrid model yields mathematically marginal gains that fail to justify its computational overhead. We attribute this to a fundamental scale mismatch: the vast spatial baseline between ICESat-2 footprints (averaging ~485 m) severely violates the dense connectivity assumptions of standard GNNs, inducing detrimental over-smoothing. Conversely, the success of the baseline XGBoost model is grounded in an explicit Physics Skeleton. By isolating deterministic drivers such as acquisition geometry (Aspect) and signal penetration (Vegetation), it proves that robustly mapping physical gradients is inherently superior to inferring implicit topologies from fragmented reference data.

(2) Second, Deep Learning Suffers from “Underspecification” in Geo-Regression.

This study provides the first quantitative evidence of the “Decoupling Hypothesis” in DEM error correction. While the Hybrid model demonstrates high predictive reproducibility (stable RMSE), it suffers from severe attribution stochasticity. Specifically, the Spearman rank correlation of its feature importance oscillates violently (ρ ≈ 0.63–0.77), compared to the near-perfect stability of the purely explicit model (ρ > 0.97). This proves that without explicit physical constraints, geometric models devolve into residual-dependent latent feature learners, capturing opportunistic spatial noise (ID Chaos) rather than discovering universal topographic laws.

(3) Finally, Explanatory Stability Must Become the New Accuracy.

Our findings serve as a critical caveat to the burgeoning Geospatial AI community: performance is not a proxy for trustworthiness. The phenomenon of “stable prediction, drifting explanation” highlights the epistemic risk of deploying unchecked “Black Box” models in physical geography. We advocate that for geospatial tasks endowed with strong physical priors, the Feature-First paradigm remains the gold standard. Moving forward, the community must pivot towards Grey Box systems (e.g., Physics-Informed Neural Networks or Graph Transformers) and institutionalize explanatory stability metrics, ensuring that AI-driven discoveries are not merely mathematically accurate, but fundamentally true to the underlying physical realities.

## Figures and Tables

**Table 1:** Main characteristics of the four tested DEMs and the reference data (ICESat-2 ATL08).

Product Name	Resolution (m)	Vertical Accuracy (m)	Acquisition Start Date	Publication Date	Datum Plain/Vertical	Spatial Bounds
ALOS World 3D (V3.2)	30	±5	2006	2021	WGS84/WGS84	84° S~84° N
Copernicus	30	±4	2011	2021	WGS84/EGM2008	85° S~84° N
SRTM GL1(V003)	30	±16	2000	2014	WGS84/WGS84	56° S~60° N
TanDEM-X	30	±2	2010	2023	WGS84/WGS84	60° S~60° N
ICESat-2 ATL08	100	0.75	2018	2020	WGS84/WGS84	90° S~90° N

Note: The ALOS World 3D and SRTM GL1 DEM products used in this study were provided in versions already converted to WGS84 ellipsoidal heights.

**Table 2:** Summary of explicit physical and geographical features utilized in the modeling phase.

Feature Type	Variable	Unit/Type	Physical Interpretation & Preprocessing
Geographic Context	Longitude & Latitude [57]	Decimal Degrees	Captures large-scale, spatially correlated systematic biases.
Absolute Terrain	Elevation [58]	Meters (m)	Source DEM elevation; absolute height often correlates with inherent sensor errors.
Local Morphometry (3 × 3 Window)	Slope [59,60]	Degrees (°)	Maximum rate of elevation change. Represents signal interaction geometry.
	Aspect (Sine & Cosine) [61]	Continuous [−1, 1]	Decomposed from raw degrees (0–360°) into two trigonometric components (Sin/Cos) to eliminate circular periodicity artifacts without information loss.
Surface Complexity (3 × 3 Window)	Interquartile Range of Slope (IQR) [62]	Continuous	A robust measure of local topographic variability, less sensitive to extreme outliers than standard deviation. Processed via log(1 + x) transformation to mitigate long-tail distribution.
	Radial Roughness Index (RRI) [63]	Continuous	Quantifies spatial entropy and terrain organization. Processed via log(1 + x) transformation.
Land Cover Attenuation	Vegetation Cover	Percentage (%)	Sourced from GFCC30TC [46]. Quantifies signal penetration limitations caused by canopy density.
	Land Cover Type [45]	Categorical	Discrete surface classes (forest, grassland, bare land, etc.). Encoded using One-Hot encoding.
	Landform Type [49]	Categorical	Geomorphological classifications (plain, platform, hills). Encoded using One-Hot encoding.

**Table 3:** Hyperparameter Search Spaces and Optimization Configuration.

Model Category	Hyperparameter	Search Space/Settings	Selection Method
XGBoost	n_estimators	[400, 1500] (step: 100)	Bayesian (Optuna)
	max_depth	[3, 10]	
	learning_rate	[0.01, 0.2] (log-scale)	
	gamma, lambda, alpha	[0, 5], [1 × 10^−8^, 1.0], [1 × 10^−8^, 1.0]	
MLP	hidden_channels	{64, 128, 256}	Bayesian (Optuna)
	n_layers	[2, 5]	
	batch_size	{512, 1024, 2048}	
Graph-based	hidden_channels	{32, 64, 128} (GraphSAGE); {64, 128} (GAT)	Bayesian (Optuna)
	heads (GAT)	{4, 8}	
	n_layers	[2, 4]	
Hybrid	GNN Encoder	Channels = 128, Layers = 3, Dropout = 0.3	Pre-defined
	XGBoost Head	Same search space as XGBoost	Two-stage Refinement
Ordinary Kriging	Variogram Model	{Linear, Power, Spherical, Exp, Gaussian}	Automated Fitting
	Parameters	Nugget, Sill, and Range	Data-driven
IDW	p (Distance Power)	{1.0, 1.5, 2.0, 2.5}	Grid Search
	k (Neighbors)	{5, 10, 15, 20}	

**Table 4:** Post-correction accuracy results of the different models on the four DEM test sets.

Method	DEM	RMSE (m)	ME (m)	NMAD (m)	MAE (m)
Original	ALOS World 3D	13.568	7.002	8.387	9.404
	Copernicus	12.945	6.349	8.099	8.817
	SRTM GL1	12.416	5.267	8.506	8.726
	TanDEM-X	13.694	6.413	8.200	9.149
XGBoost	ALOS World 3D	6.369 ± 0.004	−0.011 ± 0.003	3.513 ± 0.031	4.008 ± 0.003
	Copernicus	6.322 ± 0.002	0.023 ± 0.010	2.908 ± 0.035	3.750 ± 0.004
	SRTM GL1	6.980 ± 0.006	0.043 ± 0.002	4.820 ± 0.013	4.778 ± 0.007
	TanDEM-X	6.951 ± 0.013	0.014 ± 0.008	2.977 ± 0.029	3.974 ± 0.006
IDW	ALOS World 3D	9.061	0.060	5.769	6.139
	Copernicus	8.545	0.065	5.440	5.764
	SRTM GL1	8.913	0.138	6.266	6.239
	TanDEM-X	9.209	0.024	5.532	6.038
Kriging	ALOS World 3D	11.384	0.013	8.187	8.557
	Copernicus	10.221	0.027	7.379	7.663
	SRTM GL1	10.480	0.029	7.797	7.786
	TanDEM-X	11.747	0.011	8.914	8.728
MLP	ALOS World 3D	6.581	−0.522	3.718	4.226
	Copernicus	6.535	−0.261	2.982	3.905
	SRTM GL1	7.102	−0.547	4.937	4.910
	TanDEM-X	7.285	0.434	2.991	4.098
GraphSAGE	ALOS World 3D	6.411	0.166	3.682	4.093
	Copernicus	6.366	0.262	3.018	3.807
	SRTM GL1	6.982	0.117	4.890	4.803
	TanDEM-X	7.088	0.174	3.083	4.067
GAT	ALOS World 3D	7.793	0.713	4.706	5.135
	Copernicus	7.658	0.055	4.160	4.871
	SRTM GL1	7.982	0.039	5.555	5.539
	TanDEM-X	8.388	−0.040	4.292	5.124
GNN-XGBoost	ALOS World 3D	6.356 ± 0.002	−0.035 ± 0.003	3.546 ± 0.014	4.021 ± 0.003
	Copernicus	6.313 ± 0.002	−0.004 ± 0.010	2.920 ± 0.016	3.756 ± 0.008
	SRTM GL1	6.943 ± 0.006	0.014 ± 0.003	4.839 ± 0.014	4.765 ± 0.005
	TanDEM-X	6.975 ± 0.001	−0.022 ± 0.013	2.994 ± 0.018	3.986 ± 0.005

**Table 5:** Summary of computational cost and hardware resource utilization for the baseline and representative hybrid models.

Model	Training Time (s)	CPU Average (%)	GPU Average (MB)
XGBoost	36.86	98.31	0.00
Hybrid	666.23	50.44	236.16

Note: This table presents a summarized cost–benefit comparison between the baseline XGBoost and the representative Hybrid (k = 8) model. For the comprehensive evaluation encompassing all 7 models, various k configurations (k = 4, 16), and inference times, please refer to Table A3 in Appendix D.

**Table 6:** Statistical summary (Mean ± Std) of performance metrics for XGBoost and Hybrid models across three repeated experiments.

Method	DEM	RMSE (m)	ME (m)	NMAD (m)	MAE (m)
XGBoost	ALOS World 3D	6.369 ± 0.004	−0.011 ± 0.003	3.513 ± 0.031	4.008 ± 0.003
	Copernicus	6.322 ± 0.002	0.023 ± 0.010	2.908 ± 0.035	3.750 ± 0.004
	SRTM GL1	6.980 ± 0.006	0.043 ± 0.002	4.820 ± 0.013	4.778 ± 0.007
	TanDEM-X	6.951 ± 0.013	0.014 ± 0.008	2.977 ± 0.029	3.974 ± 0.006
GNN-XGBoost	ALOS World 3D	6.356 ± 0.002	−0.035 ± 0.003	3.546 ± 0.014	4.021 ± 0.003
	Copernicus	6.313 ± 0.002	−0.004 ± 0.010	2.920 ± 0.016	3.756 ± 0.008
	SRTM GL1	6.943 ± 0.006	0.014 ± 0.003	4.839 ± 0.014	4.765 ± 0.005
	TanDEM-X	6.975 ± 0.001	−0.022 ± 0.013	2.994 ± 0.018	3.986 ± 0.005

**Table 7:** Stability assessment of Top-10 feature importance rankings across three repeated experiments.

DEM	XGBoost ($\rho_{mean}$)	Hybrid ($\rho_{mean}$)	Interpretation of Stability
ALOS World 3D	0.992 ± 0.006	0.632 ± 0.022	Physics-Driven Stability vs. Stochastic Drift
Copernicus	0.976 ± 0.017	0.733 ± 0.013	Robust reproducibility vs. Moderate variance
SRTM GL1	0.992 ± 0.006	0.659 ± 0.070	Perfect determinism vs. Feature inconsistency
TanDEM-X	0.992 ± 0.006	0.774 ± 0.036	Physical stability vs. High explanatory stochasticity

## Data Availability

The datasets utilized and analyzed in this study were derived from the following publicly available resources: The Digital Elevation Model (DEM) data can be accessed as follows: ALOS World 3D, Copernicus DEM, and SRTM GL1 DEM are available in OpenTopography at the respective URLs: https://portal.opentopography.org/raster?opentopoID=OTALOS.082017.4326.1 (accessed on 14 September 2025), https://portal.opentopography.org/raster?opentopoID=OTSDEM.032021.4326.3 (accessed on 14 September 2025), and https://portal.opentopography.org/raster?opentopoID=OTSRTM.082016.4326.1 (accessed on 14 October 2025), as cited in references [39,40,42]. TanDEM-X EDEM is available from the German Aerospace Center (DLR) at https://download.geoservice.dlr.de/TDM30_EDEM/#details (accessed on 14 October 2025), as cited in references [6,7]. The ICESat-2 ATL08 altimetry data are available in NASA Earthdata Search at https://search.earthdata.nasa.gov/search/granules?p=C2613553260-NSIDC_CPRD&q=ATL08 (accessed on 16 October 2025), as cited in references [13,14]. The GFCC30TC vegetation data are available in NASA Earthdata Search at https://search.earthdata.nasa.gov/search?q=C2763266352-LPCLOUD (accessed on 14 October 2025), as cited in reference [45]. The GLC_FCS10 land cover data are available in Zenodo at https://zenodo.org/records/14729665 (accessed on 14 October 2025), as cited in reference [44]. The ‘1:1,000,000 scale geomorphological map of China’ data set is provided by the Geographic remote sensing ecological network platform (www.gisrs.cn, accessed on 14 October 2025), as cited in references [48].

## Appendix A

This is the detailed table of classified values of landcover types.

**Table A1:** The characteristics of the fine classification system in the GLC_FCS10.

Basic Classification System	Fine Classification System	Id
Cropland	Herbaceous rainfed cropland	11
	Tree- or shrub-covered rainfed cropland (orchard, oil palm…)	12
	Irrigated cropland	20
Forest	Closed evergreen broadleaved forest	51
	Open evergreen broadleaved forest	52
	Closed deciduous broadleaved forest	61
	Open deciduous broadleaved forest	62
	Closed evergreen needle-leaved forest	71
	Open evergreen needle-leaved forest	72
	Closed deciduous needle-leaved forest	81
	Open deciduous needle-leaved forest	82
	Closed mixed-leaf forest	91
	Open mixed-leaf forest	92
Shrubland	Evergreen shrubland	121
	Deciduous shrubland	122
Grassland	Grassland	130
Tundra	Lichens and mosses	140
Wetland	Swamp	181
	Marsh	182
	Lake/river flat	183
	Saline	184
	Mangrove forest	185
	Salt marsh	186
	Tidal flat	187
Impervious surfaces	Urban impervious surfaces	191
	Rural impervious surfaces	192
Bare areas	Sparse vegetation	150
	Bare areas	200
Water	Water	210
Permanent ice and snow	Permanent ice and snow	220

## Appendix B

This is the detailed table of landform classification values.

**Table A2:** 1:1 million spatial distribution data of terrain types in China.

Terrain Types	Id
Low-altitude plain	11
Middle-altitude plain	12
High-altitude plain	13
Extremely high-altitude plain	14
Low-altitude platform	21
Middle-altitude platform	22
High-altitude platform	23
Extremely high-altitude platform	24
Low-altitude hills	31
Middle-altitude hills	32
High-altitude hills	33
Extremely high-altitude hills	34
Minor-relief low mountains	41
Minor-relief middle mountains	42
Minor-relief high mountains	43
Minor-relief extreme mountains	44
Moderate-relief low mountains	51
Moderate-relief middle mountains	52
Moderate-relief high mountains	53
Moderate-relief extreme mountains	54
High-relief middle mountains	62
High-relief high mountains	63
High-relief extreme mountains	64
Extreme-relief middle mountains	72
Extreme-relief high mountains	73
Extreme-relief extreme mountains	74

## Appendix C

This is the sampled dataset.

## Appendix D

All experiments and timing evaluations were conducted on a workstation equipped with an Intel Xeon Gold 6226R CPU (2.90 GHz), an NVIDIA Quadro RTX 5000 GPU, and 64 GB of RAM. It is important to note that the computational costs (training/inference times) and hardware resource utilization metrics (CPU/GPU allocation) detailed in Table A3 represent a single execution cycle utilizing the optimal hyperparameter configurations. These metrics are strictly provided to establish a standardized, relative baseline for comparing the inherent algorithmic complexity and memory footprint of the evaluated models. They do not encompass the cumulative computational overhead incurred during the extensive Optuna-based hyperparameter optimization phase or the multiple independent runs required for statistical validation. Consequently, these values should be interpreted as a comparative reference for model efficiency in a deployment scenario, rather than the total computational expenditure of the entire experimental framework.

**Table A3:** Comprehensive computational cost and hardware resource utilization across all evaluated models and configurations.

Model	Training Time (s)	CPU Average (%)	GPU Average (MB)
XGBoost	36.86	98.31	0.00
MLP	172.09	5.08	16.25
IDW	2.51	1.78	17.47
Kriging	103141.55	30.83	17.47
GNN_k=4	169.66	51.89	115.20
GNN_k=8	445.92	50.59	226.28
GNN_k=16	2993.16	50.39	288.71
GAT_k=4	211.85	50.38	335.92
GAT_k=8	554.23	50.65	324.08
GAT_k=16	3237.86	47.50	348.57
Hybrid_k=4	132.19	50.44	121.96
Hybrid_k=8	666.23	50.44	236.16
Hybrid_k=16	3615.83	38.74	307.30
